# Handgrip strength reference values and dynapenia risk thresholds in an urban adult sample from Lima, Peru

**DOI:** 10.3389/fresc.2026.1904963

**Published:** 2026-08-26

**Authors:** Santos Lucio Chero-Pisfil, Aimeé Yajaira Díaz-Mau, Vanessa Elizabeth Huamanchumo-Barrientos, Rosita Maricielo Samame-Pérez, Estrella Elena Sosa-Quispe, Edgar Mario Tipula-Tipula, Ángel Eduardo Rueda-Villegas, Regina Munarriz-Medina, Antonio Esquinas

**Affiliations:** 1 Professional School of Medical Technology, Norbert Wiener University, Lima, Peru; 2 Intensive Care Unit, Hospital Meseguer, NIV-ICU Group, Biomedical Research Institute Pascual Parrilla–IMIB, Murcia, Spain

**Keywords:** aging, dynapenia, hand strength, muscle strength, reference values, sarcopenia

## Abstract

**Background:**

Handgrip strength is widely recognized as a practical biomarker of muscle function and an important screening tool for sarcopenia and functional decline. However, most di-agnostic thresholds currently used in clinical practice were developed in European or North American populations, which may limit their applicability in other ethnic groups. Evidence suggests that muscle strength varies substantially across populations, potentially leading to misclassification when universal cut-off points are applied.

**Objective:**

The objective of this study was to establish age- and sex-specific reference values for handgrip strength in Peruvian adults and to propose locally derived thresholds for identifying individuals at risk of dynapenia.

**Methods:**

A cross-sectional analytical study was conducted in 439 adults aged 20–70 years recruited from a respiratory rehabilitation center in Lima, Peru. Handgrip strength was measured using a calibrated digital dynamometer following standardized procedures recommended by the American Society of Hand Therapists. Descriptive statistics and percentile curves (p10–p90) were generated stratified by age and sex. The 10th percentile was considered a preliminary threshold for potential dynapenia. Associations between age and muscle strength were examined using Pearson correlation and simple linear regression models.

**Results:**

Men demonstrated higher mean handgrip strength than women (dominant hand: 29.97 ± 7.52 kg vs. 26.87 ± 4.66 kg; *p* < 0.005). Strength declined progressively with age, with regression models indicating an estimated reduction of approximately 2.3–2.5 kg per decade. Normative percentile distributions revealed lower absolute strength values compared with commonly used international reference standards. When contrasted with EWGSOP2 diagnostic thresholds, these differences suggested that international cut-off values may not fully reflect the physical characteristics of this population, underscoring the need for population-adapted benchmarks and further validation studies before drawing diagnostic conclusions.

**Conclusions:**

This study provides the first age- and sex-specific reference values for handgrip strength in a Peruvian adult population. The findings support the importance of population adapted thresholds for screening muscle weakness and suggest that locally derived percentiles may improve the identification of individuals at risk of functional decline in clinical and public health settings.

## 1 Introduction

Handgrip strength (HGS) is an established, non-invasive biomarker of overall physical function and neuromuscular integrity, in clinical, geriatric, and epidemiological settings, HGS serves as a vital screening tool for muscle weakness, nutritional risk, functional disability, prolonged hospital stays, and all-cause as well as cardiovascular mortality (1, 2). Major international consensus groups including the European Working Group on Sarcopenia in Older People (EWGSOP2), the Asian Working Group for Sarcopenia (AWGS 2019), the Foundation for the National Institutes of Health (FNIH) Sarcopenia Project, and the Sarcopenia Definition and Outcomes Consortium (SDOC) unanimously recognize low muscle strength as the primary operational indicator for initiating sarcopenia assessment and risk stratification (3–6). However, the translation of these consensus definitions into global practice remains constrained by universal threshold models that fail to account for population-level variability in normative muscle capacity (7).

A growing body of international evidence demonstrates that normative HGS distributions vary substantially across ethnic, geographic, socioeconomic, and anthropometric strata (7) Large-scale multinational investigations, such as the Prospective Urban Rural Epidemiology (PURE) study, data from the UK Biobank, and Asian population cohorts, have documented marked regional disparities, with absolute grip strength values in South American, African, and Southeast Asian cohorts remaining consistently lower than those recorded in Western European and North American populations (7–9). These differences are largely driven by physiological and structural factors, including average body height, hand dimensions, body proportions, and lifelong physical activity or nutritional patterns. Consequently, applying Caucasian-derived normative cut-offs to non-Western populations introduces systematic diagnostic misalignment, misclassifying healthy individuals as sarcopenic or dynapenic (10).

In Latin America, regional studies conducted in Chile, Colombia, Venezuela, and Brazil have begun to establish specific normative ranges for each population, revealing functional strength profiles determined by distinct anthropometric and socioeconomic characteristics (11–14). In Peru, previous epidemiological studies have highlighted significant discrepancies in the estimated prevalence of sarcopenia which ranges from 5.7% to 17.6% among community-dwelling older adults in the Andes, depending on whether the EWGSOP1, EWGSOP2, or FNIH criteria are applied; however, a critical review of the literature reveals that existing Peruvian studies are limited to specific geographic subregions or isolated geriatric cohorts (15). Therefore, to date, there is a complete lack of normative reference values stratified by age and sex that cover the entire adult life cycle (ages 20 to 70) and that have been derived from Peruvian urban populations.

Furthermore, although body mass index (BMI) is routinely measured in clinical practice and is often assumed to correlate with physical capacity, its precise relationship with handgrip strength remains a subject of controversy; therefore, BMI is an imperfect indicator of body composition, since individuals with identical BMI values may have radically different proportions of skeletal muscle mass, visceral fat, and muscle quality (16). In Latin American urban settings, where sarcopenic obesity is an emerging public health problem, high body mass does not inherently translate into greater voluntary contractile strength (15, 17). Therefore, an empirical evaluation is needed to clarify whether BMI acts as a linear predictor, a confounding variable, or an independent anthropometric determinant of isometric grip strength in local cohorts (18).

Crucially, an epistemological and methodological distinction must be made between *reference values* which delineate the natural descriptive distribution of muscle strength across age and sex strata and *thresholds for low muscle strength*, which serve to stratify functional risk (18). In cross-sectional designs lacking prospective tracking of hard clinical endpoints (such as falls, physical disability, rehospitalization, or mortality), thresholds derived from population percentiles (e.g., the 10th percentile of young adults) represent statistical reference benchmarks rather than clinically validated diagnostic cut-offs (11, 18). Addressing these theoretical and clinical gaps is essential for establishing context-sensitive reference frameworks tailored to local anthropometric realities (7, 10, 11).

Therefore, the primary objective of this study was to establish age- and sex-specific reference values for handgrip strength and to derive population-specific percentile-based reference thresholds for low handgrip strength in an urban clinic-based adult sample from Lima, Peru. The secondary objective was to examine the association between handgrip strength and BMI and to determine whether BMI should be considered when interpreting muscle strength in this population.

## 2 Materials and methods

### 2.1 Research design and study samples

An observational, analytical cross-sectional study was conducted in an urban adult population from Lima Metropolitana. Participant recruitment and data collection took place between May 2025 and January 2026 at a private respiratory rehabilitation center located in Lima Metropolitana, Peru. The design and reporting of the study followed the Strengthening the Reporting of Observational Studies in Epidemiology (STROBE) guidelines.

Participants were selected using a non-probabilistic convenience sampling method. Individuals who visited the center during the study period were invited to participate on a first-come, first-served basis, a strategy that may carry a potential risk of selection bias. Recruitment took place at a center in Lima specializing in cardiopulmonary physical therapy and preventive wellness. This institution offers physical training for active aging, metabolic conditioning, and medical checkups, in addition to clinical rehabilitation. To establish a representative cohort of apparently healthy adults, we recruited: community volunteers seeking preventive physical fitness evaluations; healthy family members accompanying or caring for individuals visiting the center; and the institution's administrative and clinical staff.

Initially, the eligibility of 500 prospective participants was assessed. To minimize potential selection bias, the inclusion criteria required participants to be adults of either sex, between 20 and 70 years of age, and able to understand and perform the motor instructions required for the test.

From the 500 individuals initially approached, 61 were excluded from the final analysis. Of these, 20 declined to provide written informed consent and 41 presented musculoskeletal conditions or other limiting health issues previously described in the exclusion criteria. As a result, the final analytical sample was drawn from a single private respiratory rehabilitation center and consisted of 439 apparently healthy adults, including 190 men (43.3%) and 249 women (56.7%). Participants were stratified *a priori* into five age decades. Although recruitment took place in a clinical environment, individuals with musculoskeletal, neurological, or systemic conditions affecting upper limb function were not included. This approach allowed the sample to approximate a generally healthy adult population.

### 2.2 Measurements

All measurements were performed in a single evaluation session.

#### 2.2.1 Anthropometric measurements

Anthropometric assessments were conducted under standardized conditions (after breakfast, wearing light clothing and closed footwear) and were performed by previously trained research personnel.

Body weight (kg) and height (m) were measured simultaneously using a mechanical medical column scale equipped with an integrated stadiometer (Detector®, Series 339 model, Webb City, MO, USA), with measurement accuracies of 0.1 kg for weight and 0.1 cm for height. To ensure the accuracy and reliability of the collected data, the anthropometric equipment had a valid calibration certificate issued by a metrology laboratory accredited by the National Institute of Quality of Peru. Based on these measurements, body mass index (BMI) was calculated by dividing body weight in kilograms by the square of height in meters (kg/m^2^). This calculation allowed the nutritional status of the cohort to be classified according to the current criteria established by the World Health Organization (WHO).

#### 2.2.2 Handgrip strength measurement (dynamometry)

To minimize measurement bias, all functional assessments were performed by three previously trained evaluators, ensuring a high level of inter-observer agreement. Handgrip strength (HGS) was quantified using a digital electronic dynamometer (Camry®, model EH101, Zhongshan, China). This device is equipped with a high-precision strain sensor, has a measurement capacity of up to 90 kg (198 lbs), and provides readings with a resolution of 0.1 kg, allowing accurate recording of maximal isometric grip strength.

Before each measurement, the device was reset to zero, and the assessment protocol was conducted in accordance with the standardized guidelines of the American Society of Hand Therapists (ASHT). Participants were seated on a chair without armrests, with the back upright and supported, feet flat on the floor, the shoulder adducted and in neutral rotation, the elbow flexed at 90°, and the forearm in a neutral position.

The central handle of the Camry dynamometer was individually adjusted to each participant's palmar anatomy to ensure an optimal ergonomic grip, allowing the middle phalanges of the fingers to rest comfortably on the inner handle. Participants were instructed to exert maximal grip force for 3–5 s while receiving standardized verbal encouragement from the evaluator. Three alternating trials were performed for each hand, with a 60-second recovery interval between attempts to minimize neuromuscular fatigue.

The highest value (kg) obtained across the three trials was retained for analysis because it is considered the best estimate of an individual's maximal voluntary muscle strength, while minimizing the influence of submaximal effort or trial-to-trial variability. This approach is widely used in normative handgrip strength studies and facilitates comparison with international reference values (7, 10, 19). Accordingly, maximal handgrip strength (kg), defined as the highest value obtained across the three trials, was considered the primary outcome variable of the study.

#### 2.2.3 Functional risk stratification (dynapenia)

Three clinical categories were defined based on the local percentile distribution of handgrip strength: adequate (≥ p25), at risk (p10–p25), and possible dynapenia (< p10). These locally derived thresholds were subsequently contrasted with the international diagnostic cut-off values proposed by the European Working Group on Sarcopenia in Older People (EWGSOP2) (<27 kg for men and <16 kg for women).

The primary outcome of the study was maximal handgrip strength (kg), defined as the highest value obtained across the three trials performed with either hand.

### 2.3 Statistical analysis

All statistical analyses were performed using SPSS Statistics software, version 25.0 (IBM Corp., Armonk, NY, USA). After the database cleaning process, no missing data were identified for the main study variables (muscle strength, age, and sex) among the 439 participants included in the final analysis; therefore, no statistical imputation procedures were required.

The distribution of quantitative variables was assessed using the Kolmogorov–Smirnov test.

Descriptive statistics included frequencies, means ± standard deviation (SD), and 95% confidence intervals (CI). Normative percentile curves (p10–p90) were constructed, and group differences and associations were examined using Student's t-tests, one-way analysis of variance (ANOVA), and Pearson's correlation coefficient. The rate of physiological decline in handgrip strength across age decades was estimated using simple linear regression models. In addition, the distribution of functional risk categories was analyzed using Pearson's chi-square (*χ*^2^) test and the linear-by-linear association test. Statistical significance was set at *p* < 0.05.

The 10th percentile was selected as the threshold for potential dynapenia based on previous epidemiological studies suggesting that lower decile values represent clinically meaningful reductions in muscle strength within population-based distributions.

## 3 Results

### 3.1 Sample characteristics

**Figure 1:**
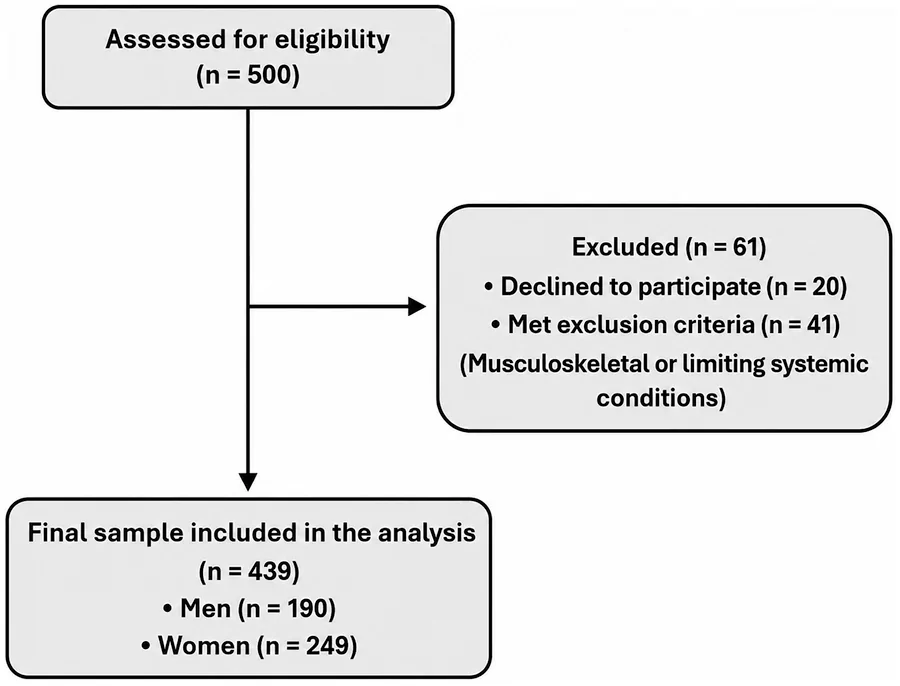
Flowchart of participant recruitment and selection. Of the 500 individuals initially assessed for eligibility, 61 were excluded (20 declined to provide informed consent and 41 met exclusion criteria related to musculoskeletal or systemic conditions), resulting in a final analytical sample of 439 participants (190 men and 249 women).

The final analytical sample comprised 439 apparently healthy adults (190 men and 249 women). The participant recruitment and selection process is summarized in Figure 1. The mean age of the study population was 38.61 ± 14.36 years. Men were significantly older than women (*p* = 0.021) and exhibited higher body weight, height, and handgrip strength in both hands (all *p* < 0.001), whereas body mass index (BMI) did not differ significantly between sexes (*p* = 0.414). Detailed baseline characteristics of the study population are presented in Table 1.

**Table 1:** General characteristics of the study population.

Characteristics	Total (*n* = 439)	Male (*n* = 190)	Female (*n* = 249)	*P*-value
Age (years)	38.61 ± 14.36	40.42 ± 15.49	37.23 ± 13.32	*p* = 0.021
Weight (kg)	67.18 ± 10.56	70.50 ± 10.27	64.65 ± 10.09	*p* < 0.005
Height (cm)	162 ± 7	166 ± 0.07	158 ± 0.05	*p* < 0.005
BMI (kg/m^2^)	25.71 ± 3.62	25.55 ± 3.45	25.83 ± 3.74	*p* = 0.414
Right hand grip strength (kg)	28,20 ± 6.25	29.97 ± 7.52	26.87 ± 4.66	*p* < 0.005
Left hand grip strength (kg)	26.41 ± 6.05	28.10 ± 7.12	25.12 ± 4.71	*p* < 0.005

General characteristics of the study population.

Data are presented as mean ± SD; N indicates the number of participants. BMI, body mass index. Statistical differences between the two groups were assessed using the unpaired Student's t-test. A value of *p* < 0.05 was considered statistically significant.

### 3.2 Reference values of handgrip strength

Age- and sex-specific handgrip strength reference values are presented in Table 2. Men consistently exhibited higher handgrip strength than women across all age groups, while both sexes showed a progressive decline with advancing age. Percentile estimates (p10, p25, p50, p75, and p90) were calculated to characterize the distribution of handgrip strength and to derive preliminary reference thresholds for functional risk stratification.

**Table 2:** Age- and sex-specific reference values for handgrip strength in healthy Peruvian adults.

Sex	Age group	n	Mean ± SD	95% CI	p10 (Risk)*	p25	p50 (Median)	p75	p90 (Optimum)
MEN	20–29	62	35.1 ± 6.2	33.5–36.6	29.1	30.9	34.7	37.6	43.9
	30–39	30	33.1 ± 5.6	31.1–35.1	27.2	28.7	32.6	35.6	40.9
	40–49	21	31.1 ± 6.0	28.5–33.6	25.7	26.2	31.0	32.8	36.2
	50–59	23	27.9 ± 7.3	24.9–30.8	18.8	22.4	25.9	32.4	38.6
	60–70	30	20.8 ± 2.2	20.0–21.6	18.0	19.3	20.8	22.3	23.6
WOMEN	20–29	89	29.2 ± 4.3	28.3–30.1	25.2	26.4	28.8	32.3	34.3
	30–39	76	28.1 ± 4.6	27.1–29.2	22.6	25.7	27.9	30.0	34.4
	40–49	28	25.7 ± 3.3	24.5–26.9	22.4	23.1	25.2	27.3	31.4
	50–59	37	23.1 ± 3.3	22.0–24.1	18.8	20.9	22.5	24.6	27.5
	60–70	26	21.3 ± 3.9	19.8–22.8	17.1	19.5	20.7	21.8	26.6

Normative reference values for handgrip strength (kg) in healthy Peruvian adults stratified by age and sex.

SD, standard deviation; CI, confidence interval. p10 corresponds to the 10th percentile and was used as the proposed screening threshold for dynapenia.

### 3.3 Age-related percentile curves of handgrip strength

**Figure 2:**
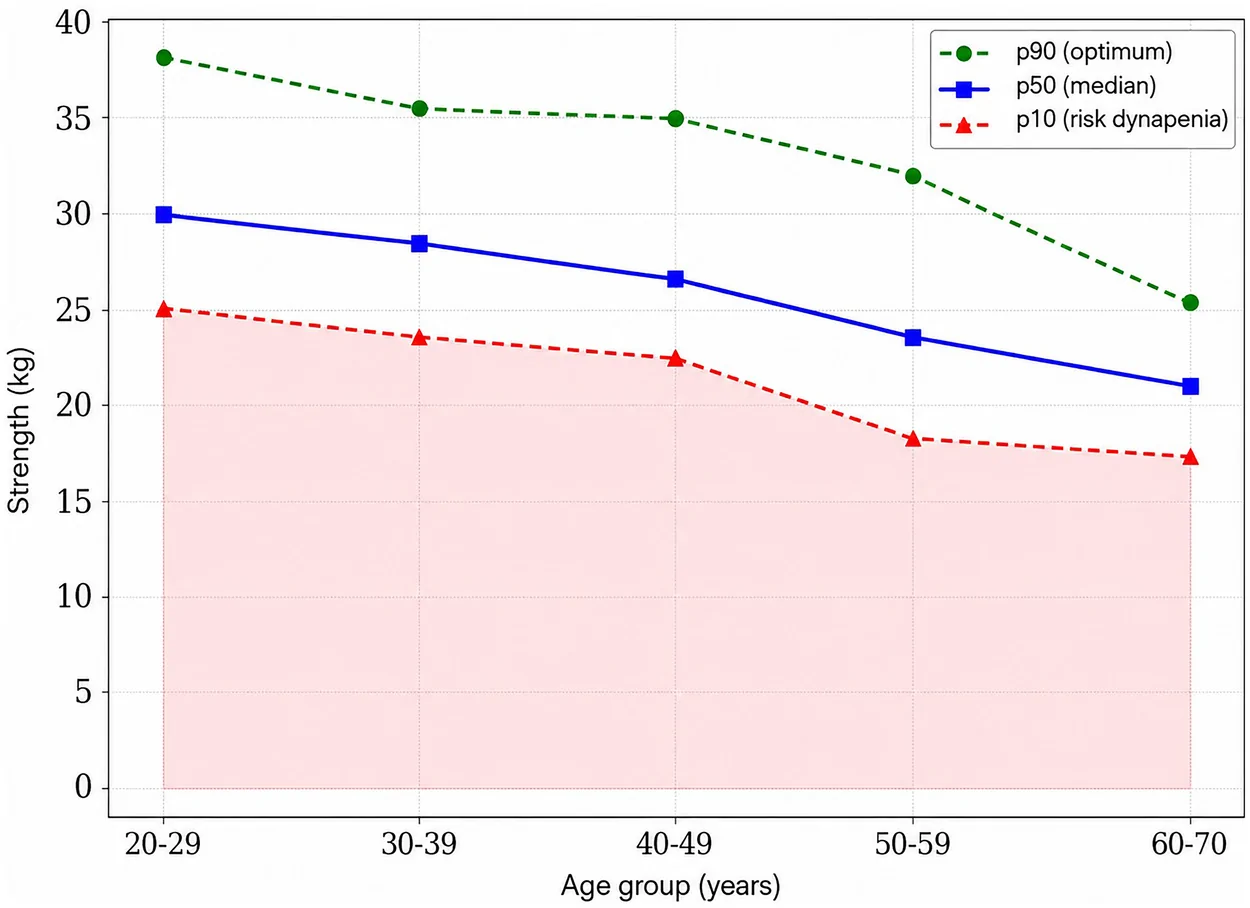
Age- and sex-specific percentile curves for handgrip strength in healthy Peruvian adults. Percentile curves (P10, P50, and P90) are presented according to 10-year age groups. The green dashed line represents the 90th percentile (optimal strength), the blue solid line represents the 50th percentile (median strength), and the red dashed line represents the 10th percentile (proposed threshold for possible dynapenia). The shaded red area indicates values below the P10 threshold.

The age- and sex-specific percentile curves revealed a gradual decline in handgrip strength across adulthood in both men and women. Men consistently exhibited higher strength values than women throughout all age groups, whereas the decline became more pronounced after the fourth decade of life. The P10, P50, and P90 curves provide a graphical representation of the distribution of handgrip strength and establish age- and sex-specific reference values for healthy Peruvian adults. Furthermore, the proposed P10 threshold offers a practical visual reference for identifying individuals with possible dynapenia in clinical and epidemiological settings (Figure 2).

### 3.4 Age-Related decline in handgrip strength

Simple linear regression demonstrated a significant inverse association between age and maximal handgrip strength for both hands (Table 3). Handgrip strength decreased by approximately 2.5 kg per decade, indicating a progressive age-related decline. The regression coefficients and model parameters are presented in Table 3.

**Table 3:** Linear regression models for predicting handgrip strength according to age and sex.

Dependent variable (handgrip strength)	Intercept (*β*0)	Slope (*β*1)*	R2	*p*-value	95% CI for β1
Right hand	34.27 kg	**−2.47 kg**	0.318	< 0.001	−2.81 a −2.13
Left hand	31.99 kg	**−2.28 kg**	0.287	< 0.001	−2.61 a −1.94

Simple linear regression models predicting handgrip strength according to age (decades) in the Peruvian adult population.

*β1 represents the estimated loss of handgrip strength (kg) per decade of life starting from age 20. CI, confidence interval.

Bold values indicate statistically significant results (*p* < 0.05).

### 3.5 Functional risk stratification according to age

**Figure 3:**
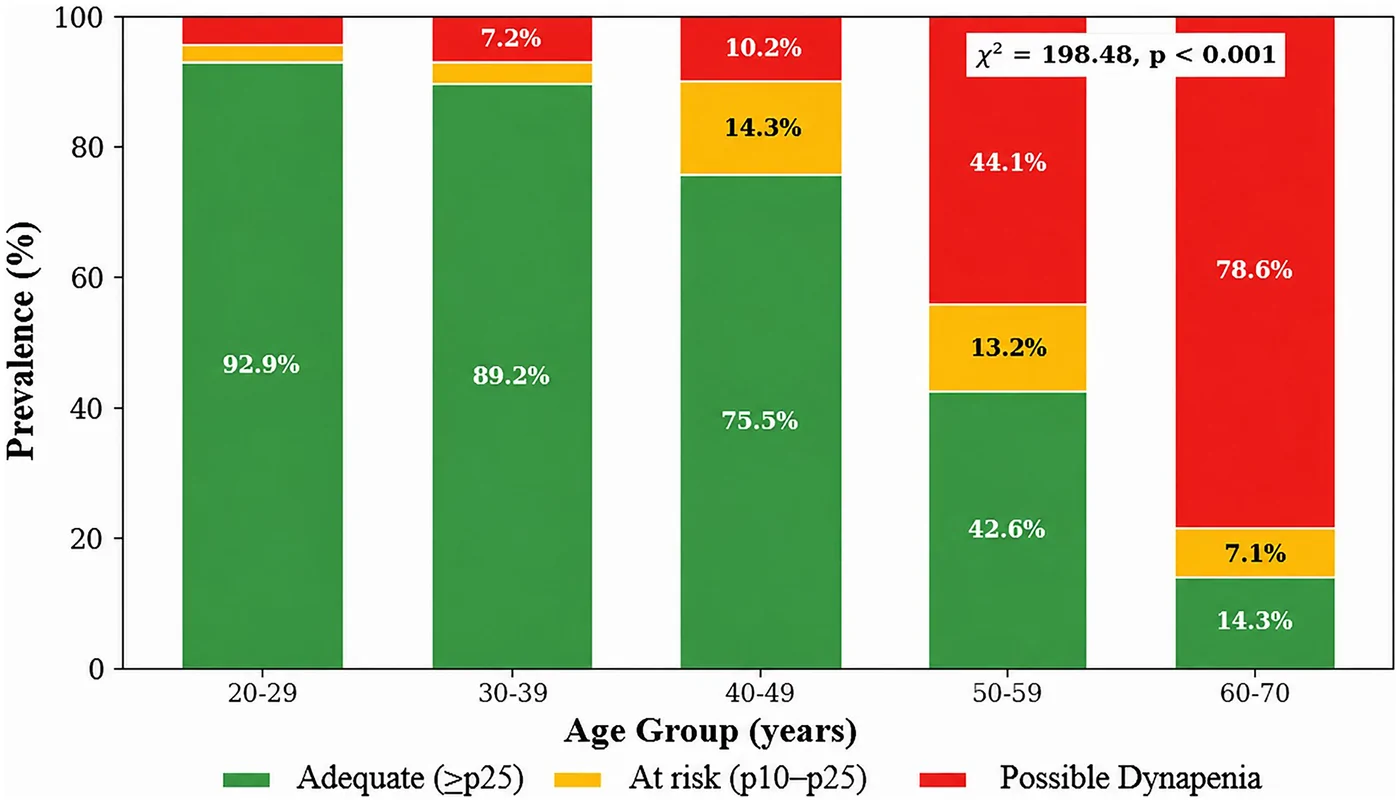
Distribution of dynapenia risk categories according to age group. Prevalence of muscle strength categories in the study population (*n* = 439). Green bars represent participants with adequate strength (≥P25), yellow bars indicate individuals at risk (P10–P25), and red bars represent possible dynapenia (<P10). The prevalence of possible dynapenia increased significantly with advancing age (*χ*^2^ = 198.48, *p* < 0.001), rising from 4.5% in participants aged 20–29 years to 78.6% in those aged 60–70 years.

The distribution of functional risk categories according to age is presented in Table 4 and Figure 3. The proportion of participants classified as having possible dynapenia increased progressively with age, whereas the prevalence of adequate muscle strength decreased significantly (*χ*^2^ = 198.48, *p* < 0.001). The highest prevalence of possible dynapenia was observed in participants aged 60–70 years.

**Table 4:** Distribution of handgrip strength categories based on percentile thresholds according to age group.

Age group	n	Adequate (≥p25)	At risk (p10–p25)	Possible dynapenia (<p10)	*p*-value*
20–29 years	155	144 (92.9%)	4 (2.6%)	7 (4.5%)	<0.001
30–39 years	111	99 (89.2%)	4 (3.6%)	8 (7.2%)	
40–49 years	49	37 (75.5%)	7 (14.3%)	5 (10.2%)	
50–59 years	68	29 (42.6%)	9 (13.2%)	30 (44.1%)	
60–70 years	56	8 (14.3%)	4 (7.1%)	44 (78.6%)	

Prevalence of functional risk categories according to age groups based on local percentile thresholds (*n* = 439).

The asterisk (*) indicates that the *p*-value was obtained using Pearson's chi-square test to assess differences in the distribution of handgrip strength categories across age groups.

### 3.6 Comparison with international diagnostic thresholds

To further explore the implications of applying international diagnostic criteria to the local population, the observed mean handgrip strength values were compared with the diagnostic cut-off proposed by the European Working Group on Sarcopenia in Older People (EWGSOP2).

**Figure 4:**
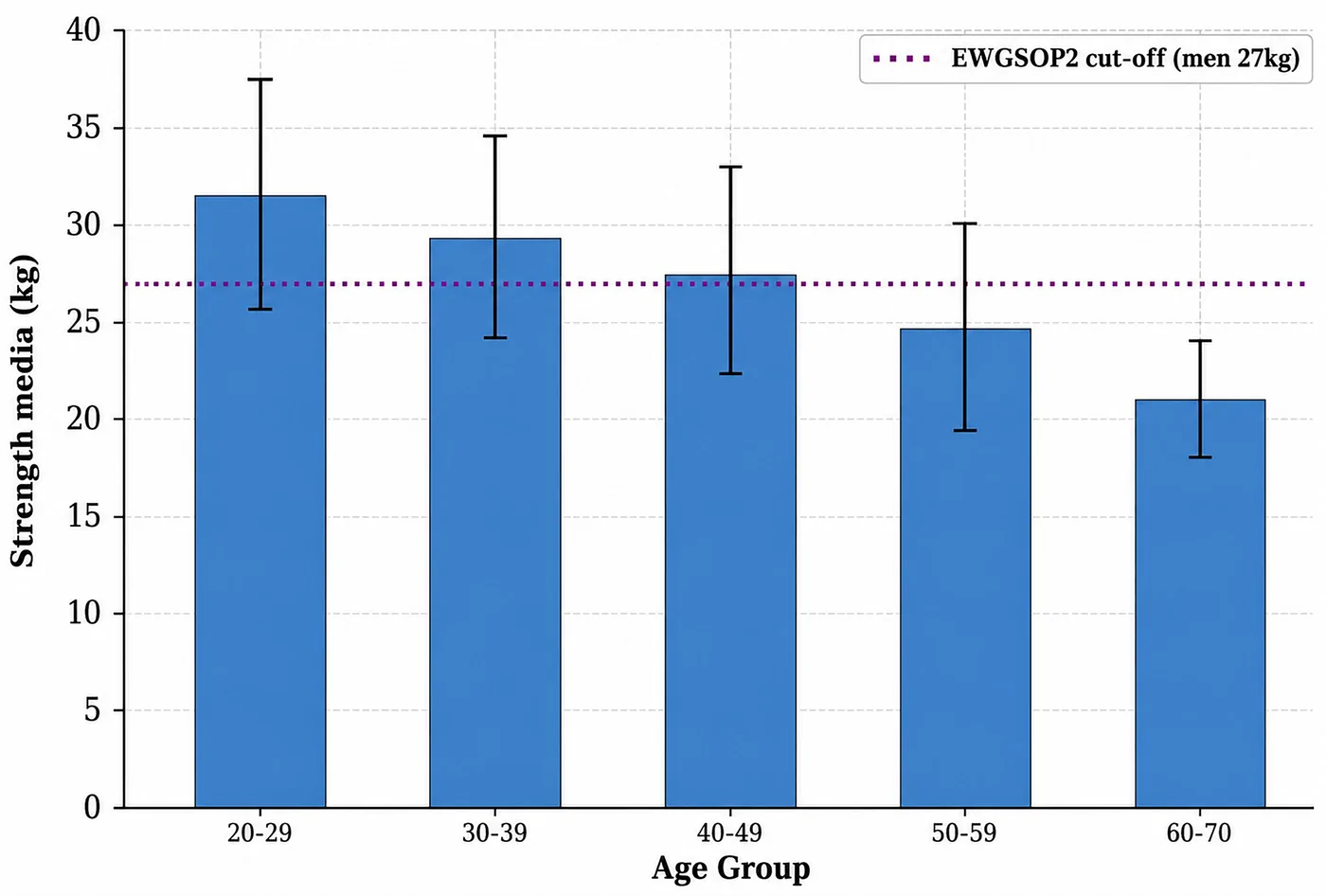
Comparison of observed handgrip strength values with the EWGSOP2 reference threshold. The dashed violet line represents the EWGSOP2 cut-off for low muscle strength in men (<27 kg). Bars represent the observed mean handgrip strength (kg) across age groups, and error bars indicate standard deviation. The figure provides a descriptive comparison between the study population and the international EWGSOP2 reference threshold and should not be interpreted as evidence of diagnostic misclassification.

The observed mean handgrip strength values were compared with the EWGSOP2 cut-off values (Table 5 and Figure 4). Mean handgrip strength decreased progressively with age, and values among older men approached or fell below the European threshold. These findings illustrate descriptive differences between the observed handgrip strength distribution in this study and the EWGSOP2 reference values; however, no conclusions regarding diagnostic performance or misclassification can be drawn from the present cross-sectional study.

**Table 5:** Comparison of observed handgrip strength values with the EWGSOP2 reference threshold.

Age group	Sex	Mean strength Peru (kg)	EWGSOP2 cut-off (kg)*	Difference (Gap)	Risk interpretation
50–59	Men	27.9	27.0	+0.9 kg	Borderline (high risk of false positives)
	Women	23.1	16.0	+7.1 kg	Adequate
60–70	Men	24.7	27.0	Negative	Potential large-scale false positive classification
	Women	21.0	16.0	+5.3 kg	Adequate

Comparison between observed mean handgrip strength in the Peruvian sample and international EWGSOP2 diagnostic thresholds.

EWGSOP2, European Working Group on Sarcopenia in Older People. Diagnostic thresholds for low muscle strength: men <27 kg; women <16 kg.

The asterisk (*) indicates the EWGSOP2 diagnostic cut-off values for low muscle strength: <27 kg for men and <16 kg for women.

## 4 Discussion

The present study establishes, for the first time, reference values for handgrip strength (HGS) in a Peruvian adult population, rigorously stratified by age and sex. The main finding reveals a population-specific strength profile, characterized by peak absolute strength values lower than those commonly reported in Caucasian reference populations, yet displaying a consistent functional decline pattern that accelerates after the fourth decade of life. Additionally, the analysis showed an absence of correlation between body mass index (BMI) and handgrip strength (r = 0.015), suggesting that body weight *per se* may not be a determinant of contractile muscle quality in this population and challenging the classical assumption of a linear relationship between body mass and muscle strength. The results presented in Table 5 and Figure 4 indicate that the uncritical application of external diagnostic cut-off points, such as those proposed by the European Working Group on Sarcopenia in Older People (EWGSOP2; <27 kg in men), may not fully reflect the physical characteristics of this cohort, emphasizing the necessity of establishing population-specific reference benchmarks before drawing diagnostic conclusions.

Our results place the mean handgrip strength in young Peruvian men (35.1 kg) below the values reported for North America and Europe (>40 kg), a discrepancy consistent with global systematic reviews that highlight the ethnic variability of handgrip strength (7). However, our findings are consistent with regional evidence, such as the PURE study (1) and recent findings in Venezuelan workers reported by Ron et al. (13), supporting the existence of a Latin American functional strength profile. These findings highlight the importance of developing population-specific reference values for muscle strength assessment in Latin America.

In this context, the lack of association observed between BMI and muscle strength is consistent with findings reported by Xiao et al. (20) in clinical settings, where recovery of body weight did not necessarily correspond to functional recovery. This observation reinforces the notion that Anthropometric assessment alone may be insufficient to determine muscle health and raises the possibility of an underlying prevalence of sarcopenic obesity, an emerging concern in the region (14, 15).

The lower absolute strength observed in this study may be partially explained by regional anthropometric characteristics, such as a lower average stature that could influence biomechanical advantage. The early decline in strength observed in this cohort is consistent with findings reported in other Latin American populations. However, the underlying mechanisms remain unclear in the Peruvian context. Future research incorporating direct measurements of inflammatory markers, oxidative stress biomarkers, and detailed body composition analysis would be necessary to determine whether systemic inflammation or other biological processes contribute to this pattern, as suggested by Kemala et al. (21).

Furthermore, as proposed by Fiut et al. (22), handgrip strength functions as an integrated biomarker of neuromuscular integrity. Therefore, the early decline observed in our cohort may reflect initial alterations in motor unit function and nerve conduction. These processes could potentially be influenced by broader social determinants of health commonly observed in developing countries, beyond the simple loss of muscle fiber mass.

The main strength of this study lies in the provision of robust local percentile curves based on a stratified sample, addressing the critical need for methodological standardization highlighted by Morin et al. (10). However, several limitations should be acknowledged. First, the cross-sectional design prevents establishing causal relationships regarding the individual rate of strength decline over time.

Additionally, although the sample is representative of an urban coastal population, it may not fully capture the genetic and environmental variability present in rural populations living at high altitude. Finally, the absence of direct measurements of inflammatory biomarkers limits our ability to confirm the specific molecular mechanisms that may underlie the early decline in muscle strength observed in this cohort.

These findings have important implications for clinical practice in Peru. The local percentile-based reference values presented in this study may serve as a foundation for future validation studies in primary care settings. However, the clinical utility of the proposed p10 threshold requires prospective validation against clinically relevant outcomes such as mortality, disability, and hospitalization.

While the diagnostic criteria proposed by the European Working Group on Sarcopenia in Older People (EWGSOP2) may require adjustment for this population, caution is recommended before replacing them until adequate local validation is completed. In this context, the values reported here should be interpreted as population-specific reference data rather than definitive diagnostic thresholds.

For future research, two priorities emerge. First, longitudinal studies are needed to validate the predictive capacity of these proposed cut-off points for clinically relevant outcomes such as mortality and physical disability. Second, further research should explore the relationship between handgrip strength and biological markers such as oxidative stress and systemic inflammation in this population, following the research line proposed by Kemala et al. (21), to better understand the biological mechanisms underlying the observed strength patterns.

A key limitation of this study is that the p10 threshold, while statistically derived, has not yet been prospectively validated against clinical outcomes. Although the percentile-based approach provides a population-specific reference framework, its clinical utility ultimately depends on demonstrating predictive validity for relevant endpoints such as mortality, functional disability, or hospitalization. Future longitudinal studies will therefore be necessary to determine whether individuals below the local p10 threshold experience worse health outcomes compared with those above it. Until such validation is completed, these values should be interpreted as reference benchmarks rather than diagnostic criteria.

Overall, these findings highlight the importance of developing population-specific reference values for muscle strength assessment in diverse populations, particularly in regions where international diagnostic thresholds may not accurately reflect local physiological characteristics.

## 5 Conclusions

This study provides the first reference values for handgrip strength in a Peruvian adult population stratified by age and sex. The findings indicate that mean strength values in this population are lower than those commonly reported in European and North American cohorts, while showing a similar age-related decline beginning after the fourth decade of life.

The percentile curves presented here may serve as a useful reference framework for evaluating muscle strength in Peruvian adults. However, the proposed p10 threshold should be interpreted cautiously until prospective studies confirm its predictive value for clinically relevant outcomes such as mortality, disability, and hospitalization. Future longitudinal research will be important to determine the clinical utility of these population-specific reference values.

## Data Availability

The raw data supporting the conclusions of this article will be made available by the authors, without undue reservation.

## References

[1] López-Bueno R Andersen LL Koyanagi A Núñez-Cortés R Calatayud J Casaña J. Thresholds of handgrip strength for all-cause, cancer, and cardiovascular mortality: a systematic review with dose-response meta-analysis. Ageing Res Rev. (2022) 82:101778. 10.1016/j.arr.2022.101778 36332759

[2] Bohannon RW. Considerations and practical options for measuring muscle strength: a narrative review. Biomed Res Int. (2019) 2019:1–10. 10.1155/2019/8194537 PMC6354207 30792998

[3] Cruz-Jentoft AJ Bahat G Bauer J Boirie Y Bruyère O Cederholm T. Sarcopenia: revised European consensus on definition and diagnosis. Age Ageing. (2019) 48:16–31. 10.1093/ageing/afy169 30312372 PMC6322506

[4] Chen L-K Woo J Assantachai P Auyeung T-W Chou M-Y Iijima K. Asian Working Group for Sarcopenia: 2019 consensus update on sarcopenia diagnosis and treatment. J Am Med Dir Assoc. (2020) 21:300–7.e2. 10.1016/j.jamda.2019.12.012 32033882

[5] Bhasin S Travison TG Manini TM Patel S Pencina KM Fielding RA. Sarcopenia definition: the position statements of the sarcopenia definition and outcomes consortium. J Am Geriatr Soc. (2020) 68:1410–8. 10.1111/jgs.16372 32150289 PMC12132920

[6] Alley DE Shardell MD Peters KW McLean RR Dam T-TL Kenny AM. Grip strength cutpoints for the identification of clinically relevant weakness. J Gerontol A Biol Sci Med Sci. (2014) 69:559–66. 10.1093/gerona/glu011 24737558 PMC3991145

[7] Dodds RM Syddall HE Cooper R Kuh D Cooper C Sayer AA. Global variation in grip strength: a systematic review and meta-analysis of normative data. Age Ageing. (2016) 45:209–16. 10.1093/ageing/afv192 26790455 PMC4776623

[8] Leong DP Teo KK Rangarajan S Kutty VR Lanas F Hui C. Reference ranges of handgrip strength from 125,462 healthy adults in 21 countries: a prospective urban rural epidemiologic (PURE) study. J Cachexia Sarcopenia Muscle. (2016) 7:535–46. 10.1002/jcsm.12112 27104109 PMC4833755

[9] Tomkinson GR Kidokoro T Dufner T Noi S Fitzgerald JS Mcgrath RP. Temporal trends in handgrip strength for older Japanese adults between 1998 and 2017. Age Ageing. (2020) 49:634–9. 10.1093/ageing/afaa021 32037458

[10] Morin M Duchesne E Bernier J Blanchette P Langlois D Hébert LJ. What is known about muscle strength reference values for adults measured by hand-held dynamometry: a scoping review. Arch Rehabil Res Clin Transl. (2022) 4:100172. 10.1016/j.arrct.2021.100172 35282144 PMC8904874

[11] Lera L Albala C Sánchez H Angel B Hormazabal MJ Márquez C. Prevalence of sarcopenia in community-dwelling Chilean elders according to an adapted version of the European working group on sarcopenia in older people (EWGSOP) criteria. J Frailty Aging. (2017) 6:12–7. 10.14283/jfa.2016.117 28244552

[12] Ramírez-Vélez R Rincón-Pabón D Correa-Bautista JE García-Hermoso A Izquierdo M. Handgrip strength: normative reference values in males and females aged 6–64 years old in a Colombian population. Clin Nutr ESPEN. (2021) 44:379–86. 10.1016/j.clnesp.2021.05.009 34330493

[13] Ron M Escalona E Ramírez-Conde F Hernández - Runque E. Validation of hand grip strength protocol: a pilot study among Venezuelan workers. Health Leadersh Qual Life. (2024) 3:e425. 10.56294/hl2024.425

[14] Pinheiro LCHT Rossi M Dos Santos CAF Oliveira LVF Vencio S de Paula Vieira R. Prevalence of associations among sarcopenia, obesity, and metabolic syndrome in Brazilian older adults. Front Med (Lausanne). (2023) 10:1206545. 10.3389/fmed.2023.1206545 37746072 PMC10514480

[15] Flores-Flores O Zevallos-Morales A Pollard SL Checkley W Siddharthan T Hurst JR. Sarcopenia and sarcopenic obesity among community-dwelling Peruvian adults: a cross-sectional study. PLoS One. (2024) 19:e0300224. 10.1371/journal.pone.0300224 38593158 PMC11003669

[16] Keevil VL Luben R Dalzell N Hayat S Sayer AA Wareham NJ. Cross-sectional associations between different measures of obesity and muscle strength in men and women in a British cohort study. J Nutr Health Aging. (2015) 19:3–11. 10.1007/s12603-014-0492-6 25560810 PMC6284799

[17] Tramontano A Veronese N Sergi G Manzato E Rodriguez-Hurtado D Maggi S. Prevalence of sarcopenia and associated factors in the healthy older adults of the Peruvian Andes. Arch Gerontol Geriatr. (2017) 68:49–54. 10.1016/j.archger.2016.09.002 27649513

[18] Ahire RSKAS. Correlation between hand grip strength and body mass index among adult women in rural area. Int J Environ Sci. (2025) 11:5491–5.

[19] Roberts HC Denison HJ Martin HJ Patel HP Syddall H Cooper C. A review of the measurement of grip strength in clinical and epidemiological studies: towards a standardised approach. Age Ageing. (2011) 40:423–9. 10.1093/ageing/afr051 21624928

[20] Xiao H Huang S Xiao H Zhang W Cai S. Longitudinal association between body mass index and handgrip strength in community-dwelling older adults: a population-based nationwide cohort study. BMC Geriatr. (2025) 25:711. 10.1186/s12877-025-06366-x 41013356 PMC12465413

[21] Kemala Sari N Stepvia S Ilyas MF Setiati S Harimurti K Fitriana I. Handgrip strength as a potential indicator of aging: insights from its association with aging-related laboratory parameters. Front Med (Lausanne). (2025) 12:1491584. 10.3389/fmed.2025.1491584 39944493 PMC11814436

[22] Fiut R Wasyluk W Świetlicka I Szukała MI Wójcik-Załuska A Dabrowski W. Muscle strength in ICU patients measured by handgrip dynamometer and ICU-acquired weakness: a prospective, single-center observational study. Med Sci Monit. (2025) 31:e949637. 10.12659/MSM.949637 41013874 PMC12490280

